# Enhanced Power Conversion Efficiency of Dye-Sensitized Solar Cells by Band Edge Shift of TiO_2_ Photoanode

**DOI:** 10.3390/molecules25071502

**Published:** 2020-03-26

**Authors:** Hye Kyeong Sung, Yeonju Lee, Wook Hyun Kim, Sang-Ju Lee, Shi-Joon Sung, Dae-Hwan Kim, Yoon Soo Han

**Affiliations:** 1 Department of Organic Material Science and Engineering, Pusan National University, Busan 46241, Korea; amrm1995@naver.com; 2 School of Advanced Materials and Chemical Engineering, Daegu Catholic University, Gyeongbuk 38430, Korea; kbg04213@naver.com; 3 Division of Energy Technology, Daegu Gyeongbuk Institute of Science and Technology (DGIST), Daegu 42988, Korea; kwh1980@dgist.ac.kr (W.H.K.); tglee@dgist.ac.kr (S.-J.L.); sjsung@dgist.ac.kr (S.-J.S.)

**Keywords:** sodium sulfide, dye-sensitized solar cell, conduction band edge shift, surface modification

## Abstract

By simple soaking titanium dioxide (TiO_2_) films in an aqueous Na_2_S solution, we could prepare surface-modified photoanodes for application to dye-sensitized solar cells (DSSCs). An improvement in both the open-circuit voltage (*V_oc_*) and the fill factor (*FF*) was observed in the DSSC with the 5 min-soaked photoanode, compared with those of the control cell without any modification. The UV–visible absorbance spectra, UPS valence band spectra, and dark current measurements revealed that the Na_2_S modification led to the formation of anions on the TiO_2_ surface, and thereby shifted the conduction band edge of TiO_2_ in the negative (upward) direction, inducing an increase of 29 mV in the *V_oc_*. It was also found that the increased *FF* value in the surface-treated device was attributed to an elevation in the shunt resistance.

## 1. Introduction

Titanium dioxide (TiO_2_, titanium (IV) oxide, or titania) has a wide range of applications such as paint, sunscreen, food coloring, photocatalysts, perovskite solar cells, and dye-sensitized solar cells (DSSCs) [1,2]. In particular, TiO_2_ photoanode is one of the important factors determining the photovoltaic properties of DSSCs. The mesoporous TiO_2_ layer provides both an extended surface area for dye adsorption and a sufficient soaking of the hole-carrying liquid electrolytes, leading to the enhanced light harvesting efficiency (LHE) and dye regeneration efficiency of DSSCs, respectively. In addition, the mesoporous TiO_2_ is an excellent recipient of photo-injected electrons from the adsorbed dyes and provides highly efficient electron transport from the injection site to the transparent electrode layer. Thus, a high short-circuit current (*J_sc_*) can be realized in DSSCs [3]. Meanwhile, it has been reported that a surface modification of the TiO_2_ layer can enhance the open-circuit voltage (*V_oc_*) due to the shift of the conduction band edge (CBE) of the TiO_2_ arising from the formation of a surface dipole. The negative shift of the TiO_2_ CBE can increase the *V_oc_* value, since it is directly proportional to the energy difference between the TiO_2_ CBE and the redox potential of the electrolyte [4]. It was reported in previous work that grafting of SrTiO_3_ [4], ZnO [5], chenodeoxycholate [6], BaCO_3_ [7], and MgO [8] onto the TiO_2_ surface shifted the CBE of TiO_2_ in the negative direction owing to the formation of a surface dipole, leading to an increase in the *V_oc_*.

However, in the previous works, the surface modification process using inorganic compounds was time and energy consuming because additional thermal annealing at an elevated temperature should be conducted after dipping TiO_2_ films in respective solutions of their precursors. In this study, we selected a strong base, i.e., sodium sulfide (Na_2_S), as a surface modifier for TiO_2_. TiO_2_ films formed on F-doped SnO_2_ (FTO)/glass substrate were immersed in an aqueous Na_2_S solution to modify their surfaces, and the resultant films were used as DSSC photoanodes. Solar cells with the Na_2_S-modified TiO_2_ layer were fabricated, and the effects of the surface modification on the photovoltaic performance were studied. We expected the TiO_2_ surface modification using strong base to alter surface charges in a short processing time, which could increase photovoltaic properties of DSSCs.

## 2. Results and Discussion

### 2.1. Modification of the TiO_2_ Surface Using an Aqueous Na_2_S Solution

In an aqueous solution, Na_2_S exists as Na^+^ and SH^−^ because sulfide (S^2−^) is more basic than hydroxide, and thus deprotonates water, as described by Equations (1) and (2). The bisulfide (SH^−^) can also react with water to produce hydroxide (OH^−^) ions (Equation (3) [9]. Two different strong bases (SH^−^ and OH^−^) exist in an aqueous Na_2_S solution (pH = 12.6), and will generate a negative charge on the TiO_2_ surface by deprotonating the surface hydroxyl groups as described by Equations (4) and (5) [10,11]. Thus, when the pristine TiO_2_/FTO was soaked in the Na_2_S solution, the TiO_2_ surface was negatively charged.

Na_2_S (s) → 2Na^+^ (aq) + S^2−^ (aq) (1)

S^2−^ (aq) + H_2_O (l) → SH^−^ (aq) + OH^−^ (aq) (2)

SH^−^ (aq) + H_2_O (l) ⇄ H_2_S (g) + OH^−^ (aq) (3)

TiO_2_-OH (s) + SH^−^ (aq) ⇄ TiO_2_-O^−^ (s) + H_2_S (g) (4)

TiO_2_-OH (s) + OH^−^ (aq) → TiO_2_-O^−^ (s) + H_2_O (l) (5)

**Figure 1:**
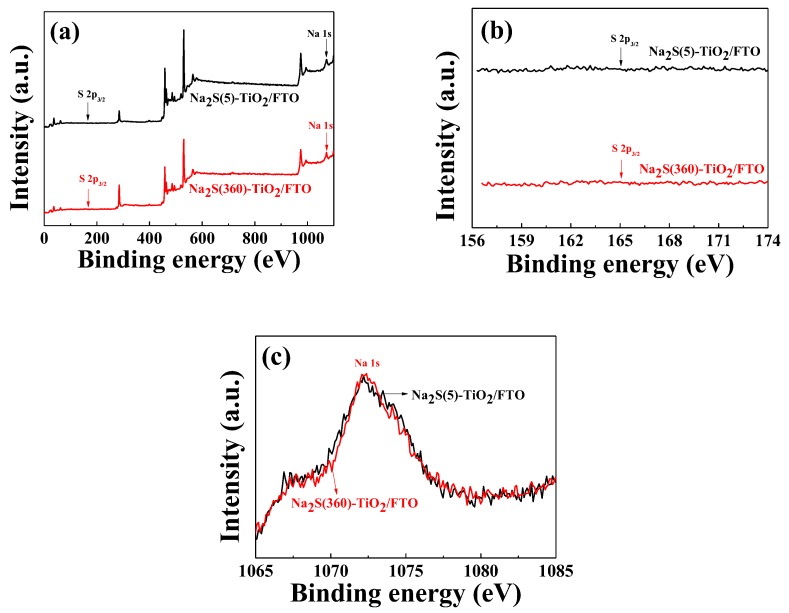
XPS spectra of the Na_2_S-TiO_2_/F-doped SnO_2_ (FTO) photoanodes; (**a**) survey scan, (**b**) S 2p_3/2_, and (**c**) Na 1s spectra.

**Figure 2:**
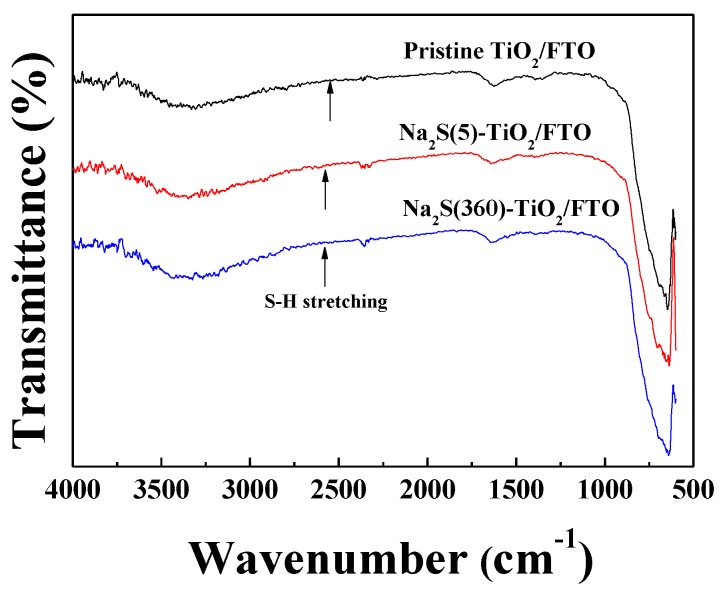
Attenuated total reflectance (ATR)-FTIR spectra of the pristine TiO_2_/FTO, the Na_2_S(5)-TiO_2_/FTO and the Na_2_S(360)-TiO_2_/FTO photoanodes.

Four different photoanodes were prepared by immersing the pristine TiO_2_/FTO in the solution for 5–20 min, thereby producing Na_2_S(5, 10, 15, and 20)-TiO_2_/FTO, where “(5)” means that the soaking time was 5 min. To characterize the TiO_2_ surface, Na_2_S(360)-TiO_2_/FTO immersed in the solution for 6 h was also prepared, and XPS measurements were performed for both the Na_2_S(5)-TiO_2_/FTO and the Na_2_S(360)-TiO_2_/FTO photoanodes. As expected from Equations (4) and (5), a sulfur peak at approximately 165 eV attributed to S 2p_3/2_ was not detected in both photoanodes, as displayed in Figure 1a,b. As a reference, it is well known that sulfonates (−SO_3_^−^) can easily adsorb on the TiO_2_ surface [12,13]. However, even with extended dipping time (6 h), sulfur was not detected on the TiO_2_ surface, indicating that bisulfide (SH^−^) was not adsorbed, and rather deprotonated the surface hydroxyl groups of TiO_2_ as shown in Equation (4). To further confirm the nonexistence of sulfur on TiO_2_ surface, the ATR-FTIR spectra were recorded for the pristine TiO_2_/FTO, the Na_2_S(5)-TiO_2_/FTO, and the Na_2_S(360)-TiO_2_/FTO photoanodes, as displayed in Figure 2. As expected, we could not observe an absorption band at 2600–2550 cm^−1^, attributed to the stretching of the S-H bond. Meanwhile, we could also observe peaks detected at 1072.9eV corresponding to the binding energy of 1s in Na in both photoanodes (Figure 1c).

### 2.2. Photovoltaic Properties of DSSCs with Na_2_S-TiO_2_/FTO

**Figure 3:**
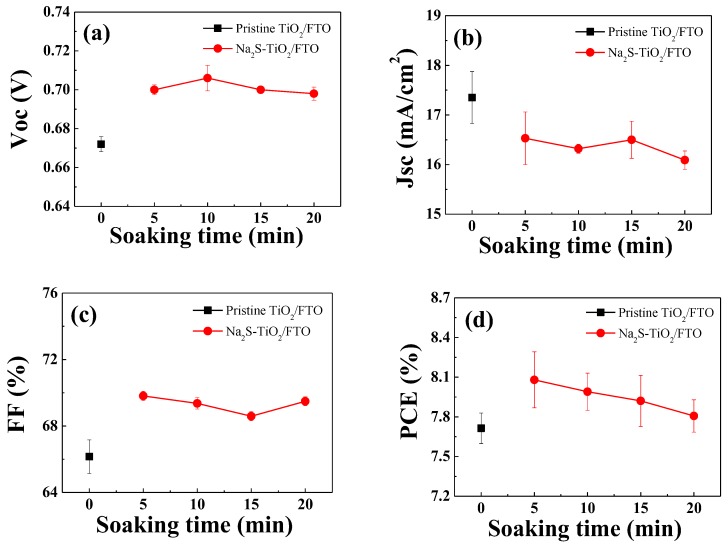
Variations in performance parameters with the soaking time in the Na_2_S solution: (**a**) *V_oc_*, (**b**) *J_sc_*, (**c**) fill factor (*FF*), and (**d**) *PCE* of the dye-sensitized solar cells (DSSCs) measured under AM 1.5 (100 mW/cm^2^) illumination.

The surface-modified photoanodes were applied in the fabrication of the DSSCs, and then their photovoltaic properties were characterized. To investigate effects of the surface modification time on the cell performance, four DSSCs were fabricated in each condition. Figure 3 shows the variations in the averaged device performance as a function of the surface modification time, namely the time of soaking in the aqueous Na_2_S solution. Detailed photovoltaic parameters are compared in Table S1 in the Supplementary Information. For all of the surface-treated DSSCs, an improved *V_oc_* was achieved compared to that of the reference cell with the pristine TiO_2_/FTO (Figure 3a). However, the *J_sc_* values decreased with increasing soaking time, as shown in Figure 3b. The fill factor (*FF*) values of the surface-treated cells were improved compared to that of the control device. Overall, the averaged power conversion efficiency (PCE) values were increased by the Na_2_S modification, because the improvement in both the *V_oc_* and the *FF* had a stronger effect on the PCE than the reduction in the *J_sc_*. 

**Figure 4:**
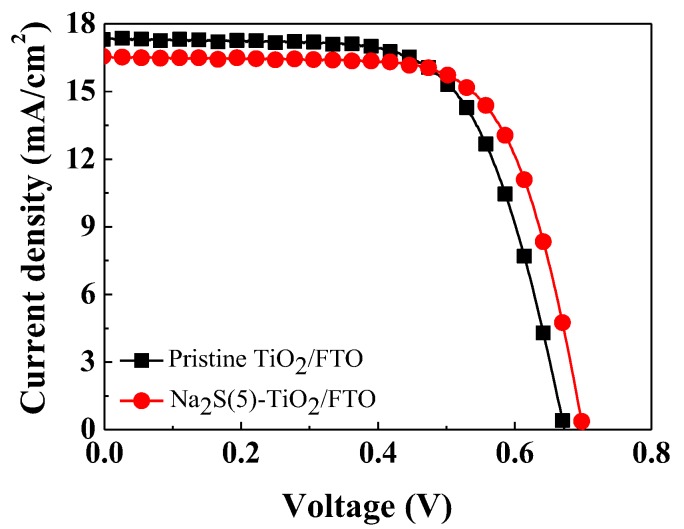
Current density–voltage (*J*–*V*) characteristics of the selected DSSCs with the pristine TiO_2_/FTO and the Na_2_S(5)-TiO_2_/FTO photoanodes.

Since the photoanode modified with the Na_2_S solution for 5 min showed the highest PCE, we focused on this device (Na_2_S(5)-TiO_2_/FTO) in our investigations of the origin of the efficiency enhancement. Among the four cells, one that showed a PCE similar to the averaged value was chosen. Photovoltaic properties of the selected cells are compared in Figure S1 and Table S2 in the Supplementary Information. The current density (*J*) and voltage (*V*) curves of the selected devices with the pristine TiO_2_/FTO and the Na_2_S(5)-TiO_2_/FTO photoanodes were compared in Figure 4, and their cell performance characteristics are listed in Table 1. The control cell with the pristine TiO_2_/FTO showed a PCE of 7.70%, while the DSSCs with the Na_2_S(5)-TiO_2_/FTO photoanode exhibited a PCE of 8.08% due to the increase in both the *V_oc_* and the *FF*. Even though the *J_sc_* of the Na_2_S(5)-TiO_2_/FTO device was lower than that of the control cell, the PCE was improved due to an increase in the *V_oc_* and the *FF*. It is important to reveal the origin of the enhancement in the *V_oc_* and the *FF*, in addition to the decrease in the *J_sc_*. 

### 2.3. Effects of Surface Modification on V_oc_

The DSSC with the Na_2_S(5)-TiO_2_/FTO exhibited 700 mV of the *V_oc_*, which was increased from that of the control cell (671 mV). This increase in the *V_oc_* is most likely due to the transformation in the energy band structure of TiO_2_ by the Na_2_S modification. To determine the electronic band structures of the photoanodes, it is necessary to measure the band gap energy (E_g_) and the valence band edge (VBE) energy, by which the CBE energy can be estimated using the equation:

CBE energy = VBE energy − E_g_ (6)

**Figure 5:**
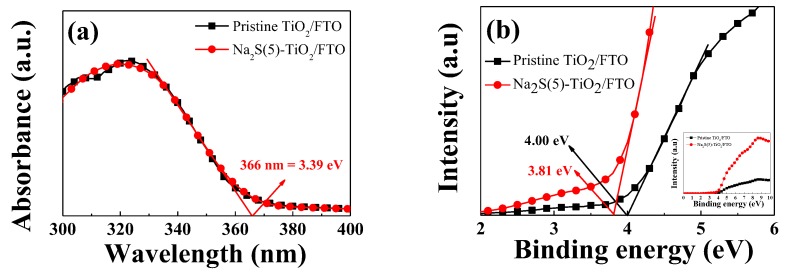
(**a**) UV–vis absorbance and (**b**) ultraviolet photoelectron spectroscopy (UPS) valence band spectra for the pristine TiO_2_/FTO and the Na_2_S(5)-TiO_2_/FTO.

**Figure 6:**
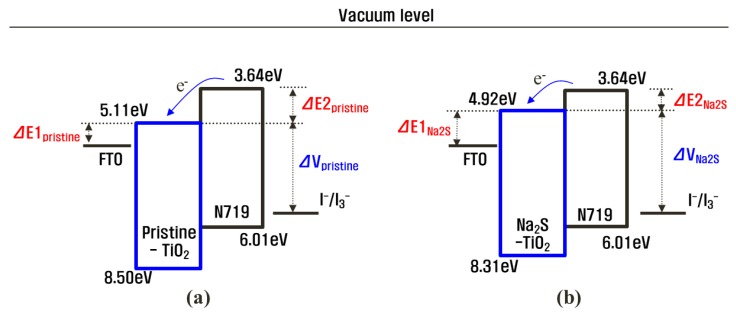
Schematic energy band diagram of (**a**) the pristine TiO_2_/FTO and (**b**) the Na_2_S(5)-TiO_2_/FTO, indicating a negative shift of the TiO_2_ conduction band edge (CBE).

We thus measured the UV–visible absorption and the UPS valence band spectra to estimate the E_g_ and the VBE energy, respectively. The absorption edge of both the pristine TiO_2_/FTO and the Na_2_S(5)-TiO_2_/FTO was observed at 366 nm, which is corresponding to an E_g_ of 3.39 eV (Figure 5a). We can also see in Figure S2a in the Supplementary Information section that the E_g_ of the Na_2_S(365)-TiO_2_/FTO was also maintained as 3.39 eV. The VBE of the pristine TiO_2_/FTO was positioned at about 4.00 eV_NHE_ (NHE = normal hydrogen electrode), while it moved to 3.81 eV_NHE_ for the Na_2_S(5)-TiO_2_/FTO, as shown in Figure 5b. The VBE of the Na_2_S(360)-TiO_2_/FTO was further shifted to a lower binding energy (i.e., 3.44 eV_NHE_), as shown in Supplementary Figure S2b. The band edge positions in NHE potential (*E*_NHE_) were converted into the energy of the absolute vacuum scale (*E*_AVS_) using the equation:

E_AVS_ = −E_NHE_ − E^0^, (7)

where *E*^0^ (approximately 4.50 eV) is the energy of the free electrons on the hydrogen scale [14,15,16]. Accordingly, the VBE energies in the pristine TiO_2_/FTO and the Na_2_S(5)-TiO_2_/FTO were calculated to be −8.50 and −8.31 eV_AVS_, respectively. Based on the identical E_g_ (3.39 eV), the CBE energies of the pristine TiO_2_/FTO and the Na_2_S(5)-TiO_2_/FTO were determined as −5.11 and −4.92 eV, respectively. As a result, the CBE in the Na_2_S(5)-TiO_2_/FTO was shifted by 19 meV in the negative direction compared to that of the pristine TiO_2_/FTO, as illustrated in Figure 6. Thus, the Na_2_S modification led to a negative shift of the TiO_2_ CBE that resulted from the presence of surface negative charges. Thus, this negative shift of the TiO_2_ CBE explains the increase in the *V_oc_* of the DSSC with the Na_2_S(5)-TiO_2_/FTO, since it induces a wider potential gap (ΔV_pristine_ < ΔV_Na2S_) between the TiO_2_ CBE and the redox potential of the electrolyte, as shown in Figure 6. Considering that the potential difference between the TiO_2_ CBE and the redox potential of the electrolyte is proportional to the *V_oc_* [17,18], it can be concluded that the increased *V_oc_* originates from the negative shift of the TiO_2_ CBE. Similar results have been reported in several previous studies that showed that the TiO_2_ surface modification using basic substances shifted the TiO_2_ CBE to a negative direction, resulting in an enhancement in *V_oc_* [10,11,19]. It is interesting that the Na_2_S modification can lead to an increase in *V_oc_* even the very short modification time of 5 min, compared with other surface modifiers studied in previous reports.

**Figure 7:**
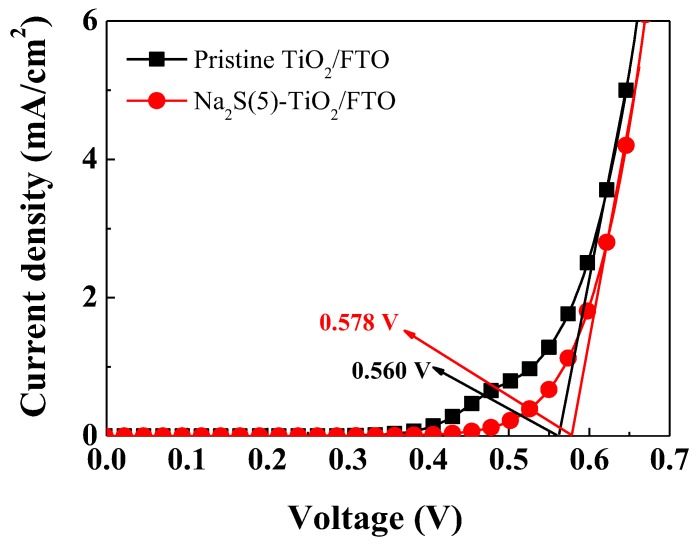
Dark current–voltage profiles of the DSSCs with the pristine TiO_2_/FTO and the Na_2_S(5)-TiO_2_/FTO photoanodes.

The dark current values of DSSCs can be used to determine a shift of the CBE [4,5]. We measured dark currents of the DSSCs with the pristine TiO_2_/FTO and the Na_2_S(5)-TiO_2_/FTO, as displayed in Figure 7. The onset potential of the dark current for the pristine TiO_2_/FTO photoanode was measured to be 0.560 V, whereas it was shifted to 0.578 V for the Na_2_S(5)-TiO_2_/FTO photoanode. Due to the Na_2_S modification, a higher onset potential was recorded in the 5 min-treated cell, implying that the potential difference between the FTO work function and the TiO_2_ CBE in the Na_2_S(5)-TiO_2_/FTO was higher than that for the pristine TiO_2_/FTO (ΔE1_pristine_ < ΔE1_Na2S_). This result suggests that the TiO_2_ CBE is shifted in the negative direction (upward direction), which is in agreement with the results of the energy band structure. 

Based on the UV–visible absorbance, UPS valence band, and dark current measurements, it was concluded that the *V_oc_* was increased by the negative shift in the TiO_2_ CBE attributed to the surface negative charges arising from the Na_2_S modification.

### 2.4. Effects of Surface Modification on J_sc_

As shown in Table 1, the Na_2_S modification induced a decrease in the *J_sc_* from 17.35 mA/cm^2^ for pristine TiO_2_/FTO to 16.53 mA/cm^2^ for Na_2_S(5)-TiO_2_/FTO. The *J_sc_* value can be expressed as

J_sc_ = ∫LHE(λ)·η_inj_(λ)·η_coll_(λ)·η_reg_·e·Φ_ph,AM1.5G_(λ) dλ (8)

where LHE(*λ*), η_inj_(*λ*), η_coll_(*λ*), and η_reg_ are the light harvesting, electron injection, electron collection, and dye regeneration efficiencies, respectively; *e* is the elementary charge and *Φ*_ph,AM1.5G_ is the photon flux in AM 1.5 G conditions of 100 mW/cm^2^ [20,21]. 

The LHE is mostly affected by the light absorbance (A) of the adsorbed dyes, that is LHE = 1 − 10^−A^ [20,21]. To estimate the effects of LHE on the *J_sc_* reduction, we measured the amount of dyes adsorbed on the surface of the TiO_2_ photoanode using the Beer–Lambert equation [22,23,24,25]. The average dye-loading amounts, as measured using five photoanodes, for the pristine TiO_2_/FTO and the Na_2_S(5)-TiO_2_/FTO were 17.39 ± 1.79 and 17.09 ± 2.61 μmol/cm^3^, respectively. The adsorbed amounts of dyes were almost identical in both photoanodes, indicating that LHE did not affect *J_sc_*. It is believed that the very short soaking time of 5 min did not influence dye-loading amounts. However, when the photoelectrodes were soaked in the Na_2_S solution more than 5 min, the *J_sc_* values were further decreased, while the *V_oc_* values were almost maintained (Figure 3). The additional decrease in the *J_sc_* is probably due to a lowered dye-loading amount in DSSCs with the Na_2_S(10, 15, and 20)-TiO_2_/FTO. At the extended soaking time, surface negative charges (i.e., TiO_2_-O^−^) would be increased, and thus they would disturb dye adsorption on TiO_2_ surface due to the repulsion between TiO_2_-O^−^ and carboxylate anions of N719 dye molecules. This could lead to an additional decrease in the *J_sc_*.

The η_inj_ value is influenced by the potential difference between the lowest unoccupied molecular orbital (LUMO) level of the dye and the TiO_2_ CBE. The measurements of the UV–visible spectra, UPS valence band spectra, and dark current revealed that the CBE of the TiO_2_ photoanode was shifted to a more negative potential by the Na_2_S modification, resulting in a smaller potential difference (ΔE2_pristine_ > ΔE2_Na2S_) between the LUMO level of the N719 dye and the TiO_2_ CBE, as illustrated in Figure 6. This can lead to a less efficient electron injection from the excited N719 dye into the conduction band of the surface-modified TiO_2_ [4,5,6,7,8]. Thus, the reduced *J*_sc_ can be attributed to the lowered η_inj_ arising from the negative (or upward) shift of the TiO_2_ CBE. 

Meanwhile, it has been reported that adsorption of various metal cations leads to the positive shift (away from the vacuum level) of the TiO_2_ CBE and thereby enhances the η_inj_ value [26,27]. However, in our study, it is believed that the positive shift of the TiO_2_ CBE by the adsorption of the sodium cations is negligible, judging from the measurements of the UV–visible spectra, UPS valence band spectra, and dark current. In view of the relatively poor signal-to-noise of the Na 1s peak (Figure 1c), it seems that a small amount of sodium was adsorbed regardless of the soaking time. It is probably because most of adsorbed sodium ions (i.e., TiO_2_-O^−^·Na^+^) were removed during rinsing with water.

**Figure 8:**
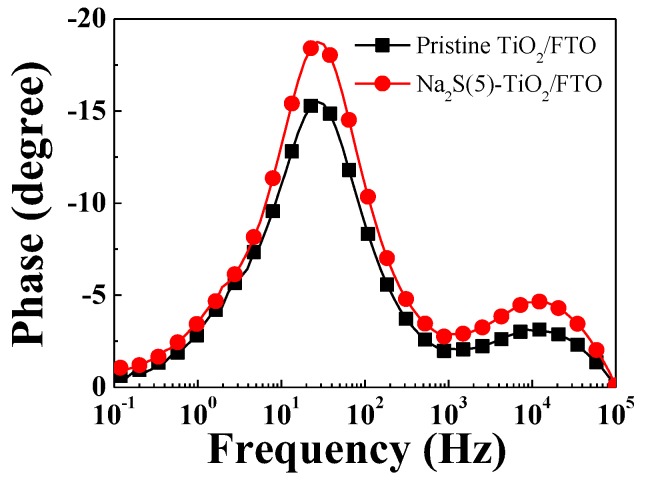
Bode plots of EIS spectra for the DSSCs with the pristine TiO_2_/FTO and the Na_2_S(5)-TiO_2_/FTO, measured under illumination (100 mW/cm^2^).

The η_coll_ value can be conjectured from the lifetime of the electrons photo-injected into the TiO_2_ valence band; that is, a prolonged electron lifetime induces an increase in η_coll_. We conducted EIS analysis to estimate the electron lifetime and charge transport of the devices with the pristine and surface-modified photoanodes [28,29,30]. As shown in the Bode phase plots of the EIS spectra (Figure 8) measured under illumination, the peak frequency (f_max_) of the device with the Na_2_S(5)-TiO_2_/FTO was obtained as 26.94 Hz, which was identical to that of the reference device. The electron lifetime (τ_n_) estimated using the equation [29,30]

τ_n_ = 1/2πf_max_, (9)

was calculated to be 5.91 ms in both devices. Thus, the unchanged electron lifetime means that η_col_ did not vary with the Na_2_S modification. This suggests that η_col_ did not affect *J_sc_*. 

Overall, it was concluded that the reduced *J_sc_* value of the DSSC with the Na_2_S(5)-TiO_2_/FTO was due to the decrease in η_inj_ resulting from the negative shift of the TiO_2_ CBE by the Na_2_S modification. 

In our previous work [31], by incorporating polyacrylonitrile, a surface dipole was formed on the TiO_2_ surface, which induced a positive shift of the TiO_2_ CBE. The positive shift resulted in an enhancement in the η_inj_, and hence the *J_sc_*. On the other hand, in this study, the TiO_2_ CBE was shifted to a negative direction by the reactions between the surface hydroxyl groups and the strong bases, leading to a decrease in the *J_sc_*. Effects of the surface modification in the two studies are totally opposite. In summary, the Na_2_S and the polyacrylonitrile act as a *V_oc_*-improver by a negative shift and as a *J_sc_*-improver by a positive shift of the TiO_2_ CBE, respectively. We can conclude that, in order to improve the *J_sc_*, surface modifiers play a role of shifting the TiO_2_ CBE to a positive direction. 

### 2.5. Effects of Surface Modification on FF

The *FF* value of the device with the 5 min-treated photoanode (69.81%) was increased compared to that of the control cell (66.16%). It is well known that the series (*R_se_*) and shunt (*R_sh_*) resistances of cells affect the *FF* value; lower *R_se_* and/or higher *R_sh_* are favorable to improve the *FF* [32,33,34]. We estimated the *R_se_* and *R_sh_* values from the slope of the *J-V* curves at *V_oc_* and *J_sc_*, respectively [32,33]. The DSSC with the Na_2_S(5)-TiO_2_/FTO had a *R_se_* of 6.05 Ωcm^2^, which was similar to that of the reference device (6.96 Ωcm^2^) with the non-treated photoanode (Table 1). The *R_sh_* value of the Na_2_S-treated DSSC was increased by 2347 Ωcm^2^ from that of the reference device (1418 Ωcm^2^). We therefore believe that the increased *R_sh_* value results in an enhancement in the *FF*. 

## 3. Experimental Details

### 3.1. Materials

For DSSC fabrication, we obtained commercial products of Solaronix (Aubonne, Switzerland) such as F-doped SnO_2_ (FTO) glass (TCO22-7) with sheet resistance of ~7 Ω/square, TiO_2_ paste for the photoanode (Ti-nanoxide T/SP), TiO_2_ paste for the scattering layer (Ti-nanoxide R/SP), N719 dye (Ruthenizer 535-bisTBA), hot-melt adhesive (SX1170-60PF, Surlyn), and iodide-based electrolytes (AN-50). Platinum paste (PT-1, acquired from Dyesol-Timo JV, Seoul, Korea) was selected as the source for the Pt counter electrode. Sodium sulfide was purchased from Sigma-Aldrich (St. Louis, MO, USA). All reagents and materials were used as received without further purification.

### 3.2. Fabrication of the DSSCs

Except for the modification process of the TiO_2_ surface, the same procedures presented in our earlier work [31] were applied to prepare DSSCs. The modification process using Na_2_S is as follows: the TiO_2_ films formed on FTO glass were soaked in an aqueous Na_2_S solution (80 mM) for 0–20 min to modify the TiO_2_ surfaces. Next, the resultant electrodes were rinsed with deionized water and ethanol, and then dried at 65 °C for 10 min to produce the modified photoanodes (Na_2_S-TiO_2_/FTO). Detailed fabrication conditions are provided in the Supplementary Information. 

### 3.3. Measurements

X-ray photoelectron spectroscopy (XPS) and ultraviolet photoelectron spectroscopy (UPS) analyses were conducted using a VG Multilab ESCA 2000 apparatus (Thermo VG Scientific, Needham, MA, USA), with Al Kα radiation (*hv* = 1486.6 eV) used for the XPS measurements and with He I radiation (*hv* = 21.22 eV) used for the UPS measurements. The energy of the C1s photoelectron peak (binding energy of 284.6 eV) was used as the reference energy. The Fourier transform infrared (FT-IR) spectra were recorded using an FT/IR 4100 spectrometer (Jasco, Tokyo, Japan) equipped with an attenuated total reflectance (ATR) accessory (PRO450-S, Jasco). The photocurrent and voltage were measured using a CompactStat potentiostat (Ivium Technologies B.V., Eindhoven, The Netherlands) and a PEC-L01 solar simulator system equipped with a 150 W xenon arc lamp (Peccell Technologies, Inc., Yokohama, Japan). To adjust light intensity to 1 sun (100 mW/cm^2^), we used a silicon photodiode (PEC-SI01, Peccell Technologies, Inc., Yokohama, Japan). The UV–Vis absorption spectra were obtained using a spectrophotometer (NEOSYS-2000, SINCO Co., Ltd., Seoul, Korea). An electrochemical analyzer (CompactStat, Ivium Technologies B.V., Eindhoven, The Netherlands) was used for electrochemical impedance spectroscopy (EIS) analysis. The active areas of the dye-sensitized TiO_2_ layers were estimated using a digital microscope camera (SZ61, OLUNPUS Corporation, Tokyo, Japan) equipped with image analysis software.

## 4. Conclusions

Na_2_S-modified TiO_2_ films were prepared by immersing TiO_2_ films into an aqueous Na_2_S solution and were applied as DSSC photoanodes. The reference device without any modification demonstrated a PCE of 7.70% (*J_sc_* = 17.35 mA/cm^2^, *V_oc_* = 671 mV, and *FF* = 66.16%), while the PCE of the cell with the Na_2_S-modified TiO_2_ layer was increased to 8.08% (*J_sc_* = 16.53 mA/cm^2^, *V_oc_* = 700 mV, and *FF* = 69.81%), due to an improvement in both the *V_oc_* and the *FF*. By the Na_2_S modification, an improvement in the *V_oc_* was achieved due to a negative shift of the TiO_2_ CBE, and the *FF* value was increased by an elevated *R_sh_*. This result indicates that Na_2_S appears to be a promising material for enhancing both the *V_oc_* and the *FF* of DSSCs.

## Figures and Tables

**Table 1:** Photovoltaic performance characteristics of the selected cells with the pristine TiO_2_/FTO and the Na_2_S(5)-TiO_2_/FTO photoanodes.

Applied Photoanodes	*V_oc_* (mV)	*J_sc_* (mA/cm^2^)	*FF* (%)	*PCE* (%)	Loaded Dye (μmol/cm^3^)	*R_se_* (Ωcm^2^)	*R_sh_* (Ωcm^2^)
Pristine TiO_2_/FTO	671	17.35	66.16	7.70	17.39	6.96	1418
Na_2_S(5)-TiO_2_/FTO	700	16.53	69.81	8.08	17.09	6.05	2347

## Supplementary Materials

The following are available online at https://www.mdpi.com/1420-3049/25/7/1502/s1, Table S1: Averages and standard deviations of cell performance, which were measured using 4 cells, with the time of soaking in the Na_2_S solution, Figure S1: *J*–*V* characteristics of selected DSSCs which showed PCEs similar to the averaged values in each soaking time, Table S2: Photovoltaic parameters of selected cells which showed PCEs similar to the averaged values in each soaking time, Figure S2: UV–vis absorbance (a) and UPS valence band (b) spectra for pristine TiO_2_/FTO, Na_2_S(5)-TiO_2_/FTO, and Na_2_S(360)-TiO_2_/FTO.

## References

[1] Haider: A.J., Jameel Z.N., Al-Hussaini I.H.M. (2019). Review on: Titanium dioxide applications. Energy Procedia.

[2] Gupta S.M., Tripathi M. (2011). A review of TiO_2_ nanoparticles. Chin. Sci. Bull..

[3] Kim J.T., Lee S.H., Han Y.S. (2015). Enhanced power conversion efficiency of dye-sensitized solar cells with Li_2_SiO_3_-modified photoelectrode. Appl. Surf. Sci..

[4] Diamant Y., Chen S.G., Melamed O., Zaban A. (2003). Core-shell nanoporous electrode for dye sensitized solar cells: The effect of the SrTiO_3_ shell on the electronic properties of the TiO_2_ core. J. Phys. Chem. B.

[5] Diamant Y., Chappel S., Chen S.G., Melamed O., Zaban A. (2004). Core-shell nanoporous electrode for dye sensitized solar cells: The effect of shell characteristics on the electronic properties of the electrode. Coord. Chem. Rev..

[6] Neale N.R., Kopidakis N., Van De Lagemaat J., Grätzel M., Frank A.J. (2005). Effect of a coadsorbent on the performance of dye-sensitized TiO_2_ solar cells: Shielding versus band-edge movement. J. Phys. Chem. B.

[7] Wu X., Wang L., Luo F., Ma B., Zhan C., Qiu Y. (2007). BaCO_3_ Modification of TiO_2_ Electrodes in Quasi-Solid-State Dye-Sensitized Solar Cells: Performance Improvement and Possible Mechanism. J. Phys. Chem. C.

[8] Bandara J., Pradeep U.W. (2008). Tuning of the flat-band potentials of nanocrystalline TiO_2_ and SnO_2_ particles with an outer-shell MgO layer. Thin Solid Films.

[9] Liao B., Wei L., Chen Z., Guo X. (2017). Na_2_S-influenced electrochemical migration of tin in a thin electrolyte layer containing chloride ions. RSC Adv..

[10] Schlichthörl G., Huang S.Y., Sprague J., Frank A.J. (1997). Band edge movement and recombination kinetics in dye-sensitized nanocrystalline TiO_2_ solar cells: A study by intensity modulated photovoltage spectroscopy. J. Phys. Chem. B.

[11] Zhang Z., Zakeeruddin S.M., O’Regan B.C., Humphry-Baker R., Grätzel M. (2005). Influence of 4-guanidinobutyric acid as coadsorbent in reducing recombination in dye-sensitized solar cells. J. Phys. Chem. B.

[12] Khazraji A.C., Hotchandani S., Das S., Kamat P.V. (1999). Controlling dye (merocyanine-540) aggregation on nanostructured TiO_2_ films. An organized assembly approach for enhancing the efficiency of photosensitization. J. Phys. Chem. B.

[13] Kim J.Y., Kim K.H., Kim D.-H., Han Y.S. (2020). Effects of a dianion compound as a surface modifier on the back reaction of photogenerated electrons in TiO_2_-based solar cells. Arabian J. Chem..

[14] Xiao G., Wang X., Li D., Fu X. (2008). InVO_4_-sensitized TiO_2_ photocatalysts for efficient air purification with visible light. J. Photochem. Photobiol. A Chem..

[15] Sun L., Qi Y., Jia C.J., Jin Z., Fan W. (2014). Enhanced visible-light photocatalytic activity of g-C_3_N_4_/Zn_2_GeO_4_ heterojunctions with effective interfaces based on band match. Nanoscale.

[16] Zhang J., Ren F., Deng M., Wang Y. (2015). Enhanced visible-light photocatalytic activity of a g-C_3_N_4_/BiVO_4_ nanocomposite: A first-principles study. Phys. Chem. Chem. Phys..

[17] Chen S., Lin J., Wu J. (2014). Facile synthesis of Y_2_O_3_:Dy^3+^ nanorods and its application in dye-sensitized solar cells. Appl. Surf. Sci..

[18] Nath N.C.D., Lee J.J. (2019). Binary redox electrolytes used in dye-sensitized solar cells. J. Ind. Eng. Chem..

[19] Kim C., Kim J.T., Kim H., Park S.H., Son K.C., Han Y.S. (2010). Effects of metal hydroxide-treated photoanode on the performance of hybrid solar cells. Curr. Appl. Phys..

[20] Hagfeldt A., Boschloo G., Sun L., Kloo L., Pettersson H. (2010). Dye-Sensitized Solar Cells. Chem. Rev..

[21] Arkan F., Izadyar M., Nakhaeipour A. (2016). The role of the electronic structure and solvent in the dye-sensitized solar cells based on Zn-porphyrins: Theoretical study. Energy.

[22] Park J.T., Roh D.K., Chi W.S., Patel R., Kim J.H. (2012). Fabrication of double layer photoelectrodes using hierarchical TiO_2_ nanospheres for dye-sensitized solar cells. J. Ind. Eng. Chem..

[23] Alarcón H., Hedlund M., Johansson E.M.J., Rensmo H., Hagfeldt A., Boschloo G. (2007). Modification of Nanostructured TiO_2_ Electrodes by Electrochemical Al^3+^ Insertion: Effects on Dye-Sensitized Solar Cell Performance. J. Phys. Chem. C.

[24] Lee K.E., Gomez M.A., Charbonneau C., Demopoulos G.P. (2012). Enhanced surface hydroxylation of nanocrystalline anatase films improves photocurrent output and electron lifetime in dye sensitized solar cell photoanodes. Electrochim. Acta.

[25] Kim J.T., Han Y.S. (2014). Effects of surface-modified photoelectrode on the power conversion efficiency of dye-sensitized solar cells. Met. Mater. Int..

[26] Kelly C.A., Farzad F., Thompson D.W., Stipkala J.M., Meyer G.J. (1999). Cation-controlled interfacial charge injection in sensitized nanocrystalline TiO_2_. Langmuir.

[27] Tachibana Y., Haque S.A., Mercer I.P., Moser J.E., Klug D.R., Durrant J.R. (2001). Modulation of the rate of electron injection in dye-sensitized nanocrystalline TiO_2_ films by externally applied bias. J. Phys. Chem. B.

[28] Kim J.Y., Kwak G., Choi Y.C., Kim D.-H., Han Y.S. (2019). Enhanced performance of perovskite solar cells by incorporation of a triphenylamine derivative into hole-transporting poly(3-hexylthiophene) layers. J. Ind. Eng. Chem..

[29] Kim K.S., Song H., Nam S.H., Kim S.-M., Jeong H., Kim W.B., Jung G.Y. (2012). Fabrication of an Efficient Light-Scattering Functionalized Photoanode Using Periodically Aligned ZnO Hemisphere Crystals for Dye-Sensitized Solar Cells. Adv. Mater..

[30] Zhao J., Sun B., Qiu L., Caocen H., Li Q., Chen X., Yan F. (2012). Efficient light-scattering functionalized TiO_2_ photoanodes modified with cyanobiphenyl-based benzimidazole for dye-sensitized solar cells with additive-free electrolytes. J. Mater. Chem..

[31] Baek G.W., Kim Y.-J., Jung K.-H., Han Y.S. (2019). Enhancement of solar cell performance through the formation of a surface dipole on polyacrylonitrile-treated TiO_2_ photoelectrodes. J. Ind. Eng. Chem..

[32] Lü X., Mou X., Wu J., Zhang D., Zhang L., Huang F., Xu F., Huang S. (2010). Improved-performance dye-sensitized solar cells using Nb-doped TiO_2_ electrodes: Efficient electron injection and transfer. Adv. Funct. Mater..

[33] Koide N., Islam A., Chiba Y., Han L. (2006). Improvement of efficiency of dye-sensitized solar cells based on analysis of equivalent circuit. J. Photochem. Photobiol. A.

[34] Syrrokostas G., Leftheriotis G., Yianoulis P. (2014). Effect of acidic additives on the structure and performance of TiO_2_ films prepared by a commercial nanopowder for dye-sensitized solar cells. Renew. Energy.

