# Supplementary material for: Enhanced Power Conversion Efficiency of Dye-Sensitized Solar Cells by Band Edge Shift of TiO2 Photoanode
Source: Molecules. 2020 Mar 26;25(7):1502. doi: 10.3390/molecules25071502 (PMC7181208; doi:10.3390/molecules25071502)
Supplement: Supplementary file 1 [file molecules-25-01502-s001.pdf]

molecules

Supplementary Information

Enhanced Power Conversion Efficiency of Dye-Sensitized Solar Cells by Band Edge Shift of TiO_2 Photoanode

Hye Kyeong Sung [1,†], Yeonju Lee [2,†], Wook Hyun Kim [3], Sang-Ju Lee [3], Shi-Joon Sung [3], Dae-Hwan Kim [3,*] and Yoon Soo Han [2,]**

[1] Department of Organic Material Science and Engineering, Pusan National University, Busan 46241, Korea; amrm1995@naver.com
[2] School of Advanced Materials and Chemical Engineering, Daegu Catholic University, Gyeongbuk 38430, Korea; kbg04213@naver.com
[3] Division of Energy Technology, Daegu Gyeongbuk Institute of Science and Technology (DGIST), Daegu 42988, Korea; kwh1980@dgist.ac.kr (W.H.K.); tglee@dgist.ac.kr (S.-J.L.); sjsung@dgist.ac.kr (S.-J.S.)
* Correspondence: monolith@dgist.ac.kr (D.-H.K.); yshancu@cu.ac.kr (Y.S.H.); Tel.: (+82)53-785-3720 (D.-H.K.); (+82)53-850-2773 (Y.S.H.); Fax: (+82)53-785-3739 (D.-H.K.); (+82)53-359-6662 (Y.S.H.)
† These authors contributed equally to this work.

1. Fabrication Conditions of DSSCs

The FTO glass samples were sonicated for 20 min in deionized water (DIW), acetone, and ethyl alcohol, and were then thoroughly rinsed with DIW and ethanol. The cleaned FTO glasses were dipped in a hydrolyzed $TiCl_4$ solution (40 mM) at 70°C for 30 min, and then washed with DIW and ethanol. A TiO_2 (15–20 nm) paste was coated on the $TiCl_4$-treated FTO glass via doctor-blade method, and successively a TiO_2 layer composed of particles with a diameter of over 100 nm was deposited. The two layers were then sintered at 500°C for 60 min to produce both the mesoporous and scattering TiO_2 layers. Finally, the TiO_2 films were again treated with a hydrolyzed $TiCl_4$ solution (40 mM) and annealed at 500 °C for 60 min. Thus, TiO_2/FTO photoanodes with scattering layers were prepared. The anodes were soaked in an aqueous Na_2S solution (80 mM) for 0–20 min to modify the TiO_2 surfaces. Next, the resultant electrodes were rinsed with DIW and ethanol, and then dried at 65°C for 10 min to produce the modified photoanodes (Na_2S-TiO_2/FTO). Finally, both the pristine TiO_2/FTO and the Na_2S-TiO_2/FTO photoanodes were sensitized by separately dipping them in a 0.5 mM ethanolic N719 dye solution for 24 h to obtain the working electrodes.

For the preparation of the counter electrode, the FTO glasses were drilled to form two holes, and then cleaned by ultrasonication as described above. A Pt paste was deposited via the doctor-blade method on the cleaned FTO glasses and followed by sintering at 400 °C for 30 min to form Pt catalyst layers. The prepared counter and working electrodes were sealed using a 60-μm-thick hot melt adhesive (Surlyn). The electrolyte was injected into the cells through one of the two small holes drilled into the counter electrodes to produce DSSCs with an active area of 25 mm^2.

2. Averaged Photovoltaic Performance of DSSCs

Table S1. Averages and standard deviations of cell performance, which were measured using 4 cells, with the time of soaking in the Na_2S solution.

Soaking time (min)	V_{oc} (mV)	J_{sc} (mA/cm^2)	*FF* (%)	η (%)
0	0.675 ± 0.004	17.04 ± 0.523	67.28 ± 1.006	7.73 ± 0.115
5	0.697 ± 0.002	16.69 ± 0.530	69.66 ± 0.002	8.10 ± 0.212
10	0.697 ± 0.007	16.21 ± 0.090	69.14 ± 0.342	7.81 ± 0.141
15	0.699 ± 0.002	15.99 ± 0.371	68.76 ± 0.279	7.68 ± 0.193
20	0.693 ± 0.003	15.98 ± 0.185	69.12 ± 0.271	7.66 ± 0.122

3. Current Density-Voltage Profiles of DSSCs

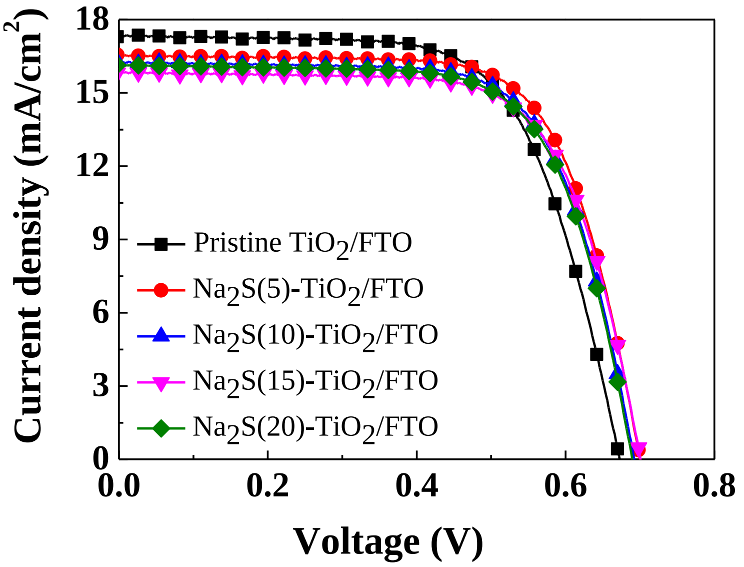

Figure S1. *J–V* characteristics of selected DSSCs which showed PCEs similar to the averaged values in each soaking time.

Table S2. Photovoltaic parameters of selected cells which showed PCEs similar to the averaged values in each soaking time.

Applied photoanodes	V_{oc} (mV)	J_{sc} (mA/cm^2)	*FF* (%)	η (%)	R_{se} (Ωcm^2)	R_{sh} (Ωcm^2)
Pristine TiO_2/FTO	671	17.35	66.16	7.70	6.96	1418
Na_2S(5)-TiO_2/FTO	700	16.53	69.81	8.08	6.05	2347
Na_2S(10)-TiO_2/FTO	692	16.21	69.41	7.79	6.32	2169
Na_2S(15)-TiO_2/FTO	700	15.86	69.15	7.68	6.46	1835
Na_2S(20)-TiO_2/FTO	690	16.13	68.85	7.66	6.20	2375

4. UV–Vis Absorbance and UPS Valence Band Spectra

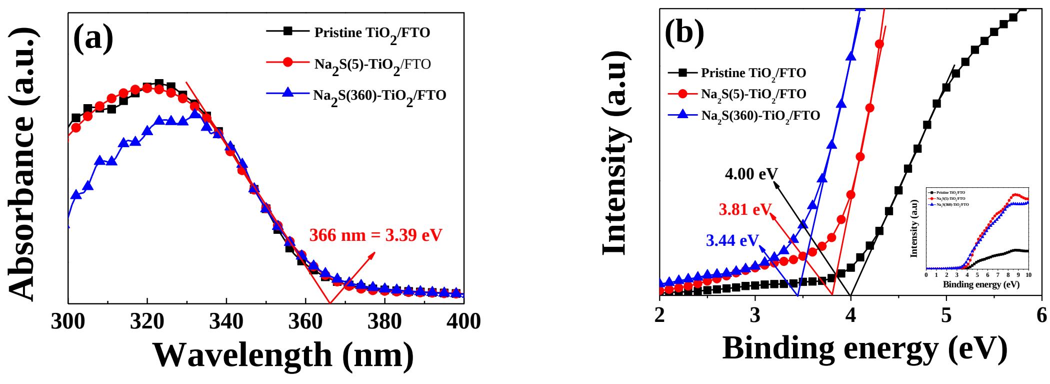

Figure S2. UV–vis absorbance (a) and UPS valence band (b) spectra for pristine TiO_2/FTO, Na_2S(5)-TiO_2/FTO, and Na_2S(360)-TiO_2/FTO.

© 2020 by the authors. Submitted for possible open access publication under the terms and conditions of the Creative Commons Attribution (CC BY) license (http://creativecommons.org/licenses/by/4.0/).
